# Supplementary material for: Macrophage achieves self-protection against oxidative stress-induced ageing through the Mst-Nrf2 axis
Source: Nat Commun. 2019 Feb 14;10:755. doi: 10.1038/s41467-019-08680-6 (PMC6376064; doi:10.1038/s41467-019-08680-6)
Supplement: Supplementary file 1 — Supplementary Information [file 41467_2019_8680_MOESM1_ESM.pdf]

Supplementary Information for

Macrophage achieves self-protection against oxidative stress-induced ageing through the Mst-Nrf2 axis

Wang P. *et al*.

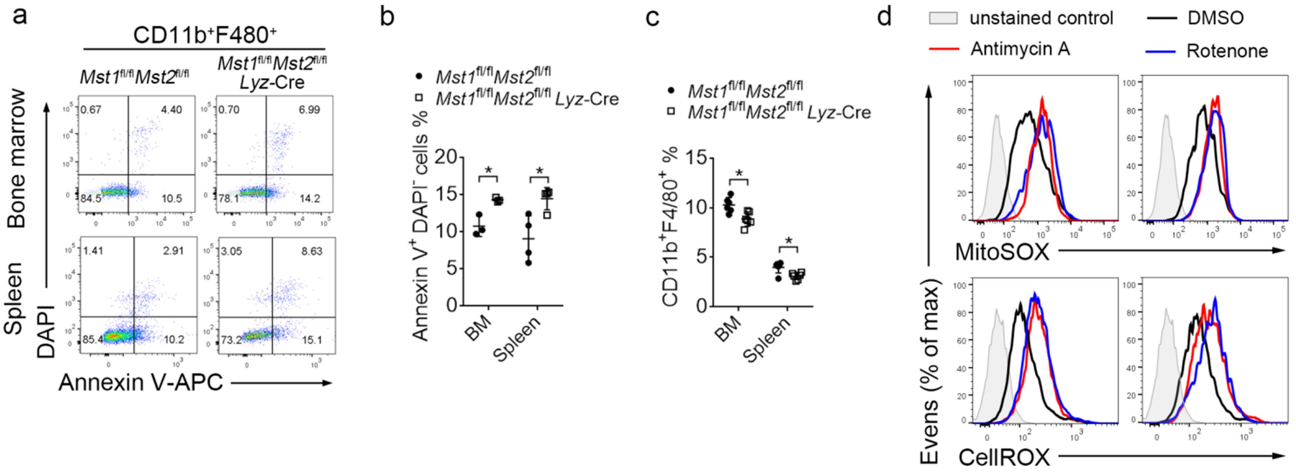

Supplementary Figure 1. Increase ROS levels and apoptosis events in Mst1/2 null macrophages.

(a-c) Annexin V/DAPI staining (a) and quantification of Annexin V^+DAPI^- cells (b) and total cell number (c) of $CD11b^+F4/80^+$ cells in spleen and bone marrow of *Mst1*$^{fl/fl}$*Mst2*$^{fl/fl}$ (WT) and *Mst1*$^{fl/fl}$*Mst2*$^{fl/fl}$ *Lyz*-Cre (DKO) mice. *P < 0.05 compared between WT and DKO groups (Student's t-test, mean and s.d. of n = 4, biologically independent animals). (d) Flow cytometry analysis of mitochondrial and cellular ROS levels in WT BMDMs treated with antimycin A or rotenone, followed by MitoSOX or CellROX staining respectively for 30 min. Data are from one experiment representative of three independent experiments with similar results.

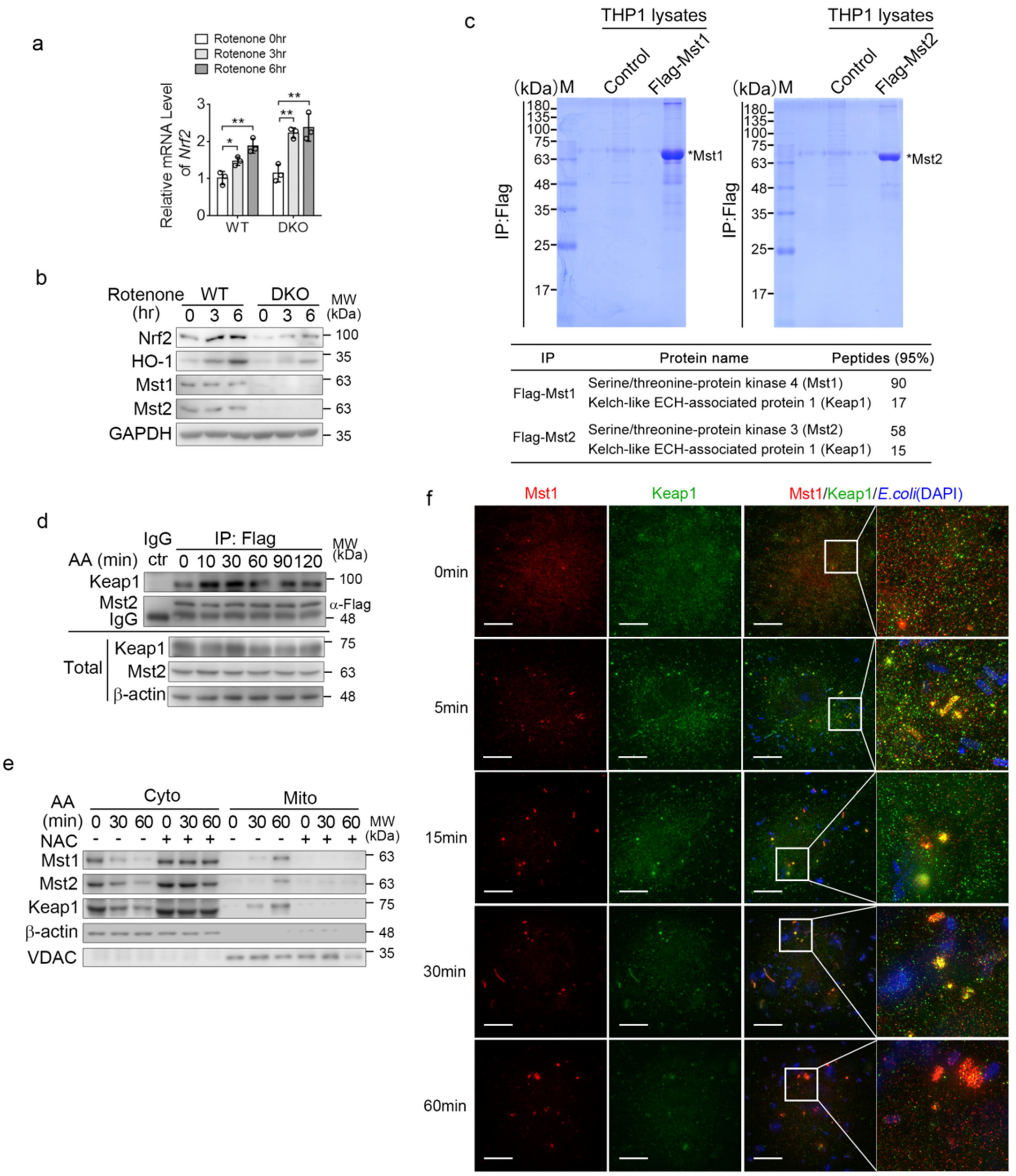

Supplementary Figure 2. Interaction of Mst1/2 and Keap1.

(a and b) RT-qPCR analysis of *Nrf2* (a) and immunoblot analysis of Nrf2, HO-1, Mst1, Mst2 and GAPDH (b) in *Mst1*$^{fl/fl}$*Mst2*$^{fl/fl}$ (WT) and *Mst1*$^{fl/fl}$*Mst2*$^{fl/fl}$ *Lyz*-Cre (DKO) BMDMs treated with 3 μM rotenone for indicated times. *P < 0.05 and **P < 0.01, compared with the control sample (Student's t-test, mean and s.d. of n=3, experimental replicates). (c) Identification and the list of Keap1 and Mst1 or Mst2 by mass spectrometry in a Flag-tagged Mst1- or Mst2-precipitation assay in THP1 cell lysates. (d) Immunoblot analysis of Keap1 and Mst2 in anti-IgG or anti-Flag IP and total lysates of WT BMDM cell line stably expressing Flag-tagged Mst2 treated with antimycin A (AA) for indicated times. (e) Immunoblot analysis of Mst1, Mst2, β-actin and VDAC in the cytoplasmic (Cyto) and mitochondrial (Mito) fractions of WT BMDMs pretreated with or without NAC and followed by AA treatment for indicated times. (f) SIM of co-localization of Mst1 (red) and Keap1 (green) in WT BMDMs infected with E. coli (blue) for the indicated time; 16× magnification of areas outlined in the main images are shown next to the main images. Scale bars, 20 μm.

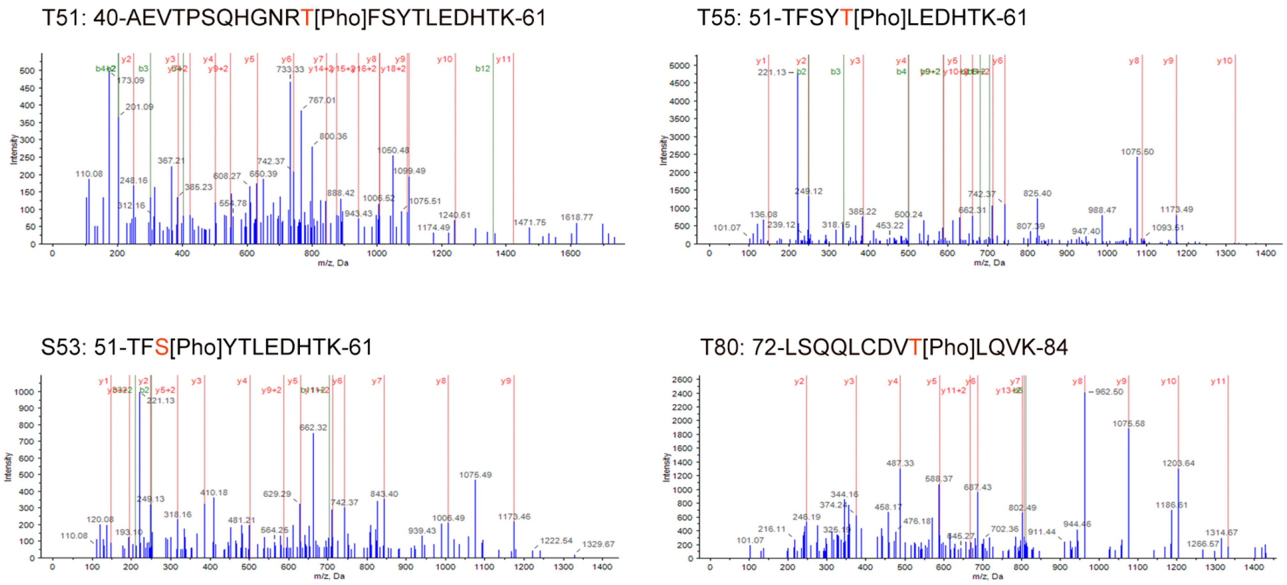

Supplementary Figure 3. Identification of Mst1/2-mediated Keap1 phosphorylation sites. Phosphorylation sites of Keap1 were detected by mass spectrometry analysis of bacterially expressed GST-tagged Keap1 from an in vitro kinase reaction with HA-tagged Mst2.

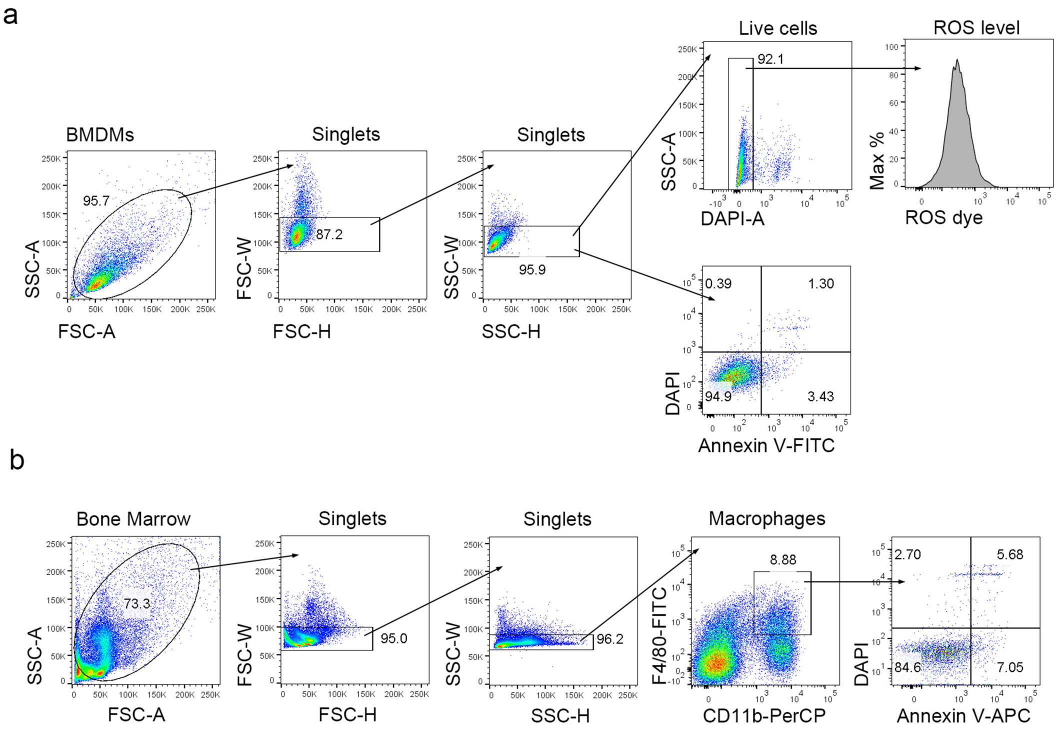

Supplementary Figure 4. Flow cytometry gating strategy for ROS and apoptosis measurements.

(a) Gating strategy for the ROS or apoptosis measurement of BMDMs or THP1 cells presented on Fig. 1a, e and j, Fig. 3a and l, Fig. 4h and I, Fig. 8a and Supplementary Fig. 1d.

(b) Representative flow cytometry dot plots and the employed gating strategy for apoptosis analysis in bone marrow CD11b$^+$F4/80$^+$ macrophages on Fig. 1g and Supplementary Fig. 1a.

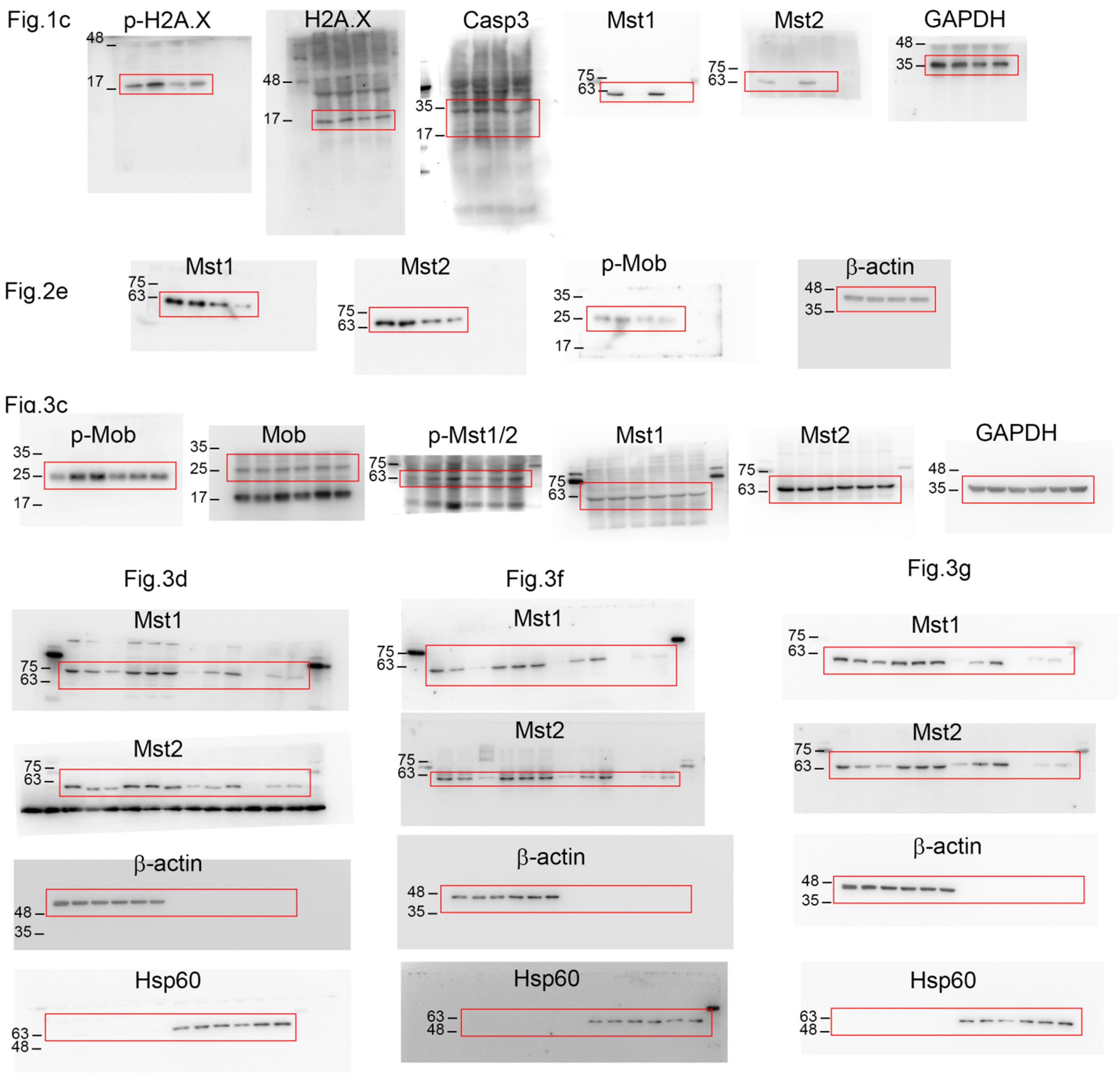

Supplementary Figure 5. Full scans for Fig. 1c, 3c, 3d, 3f and 3g.

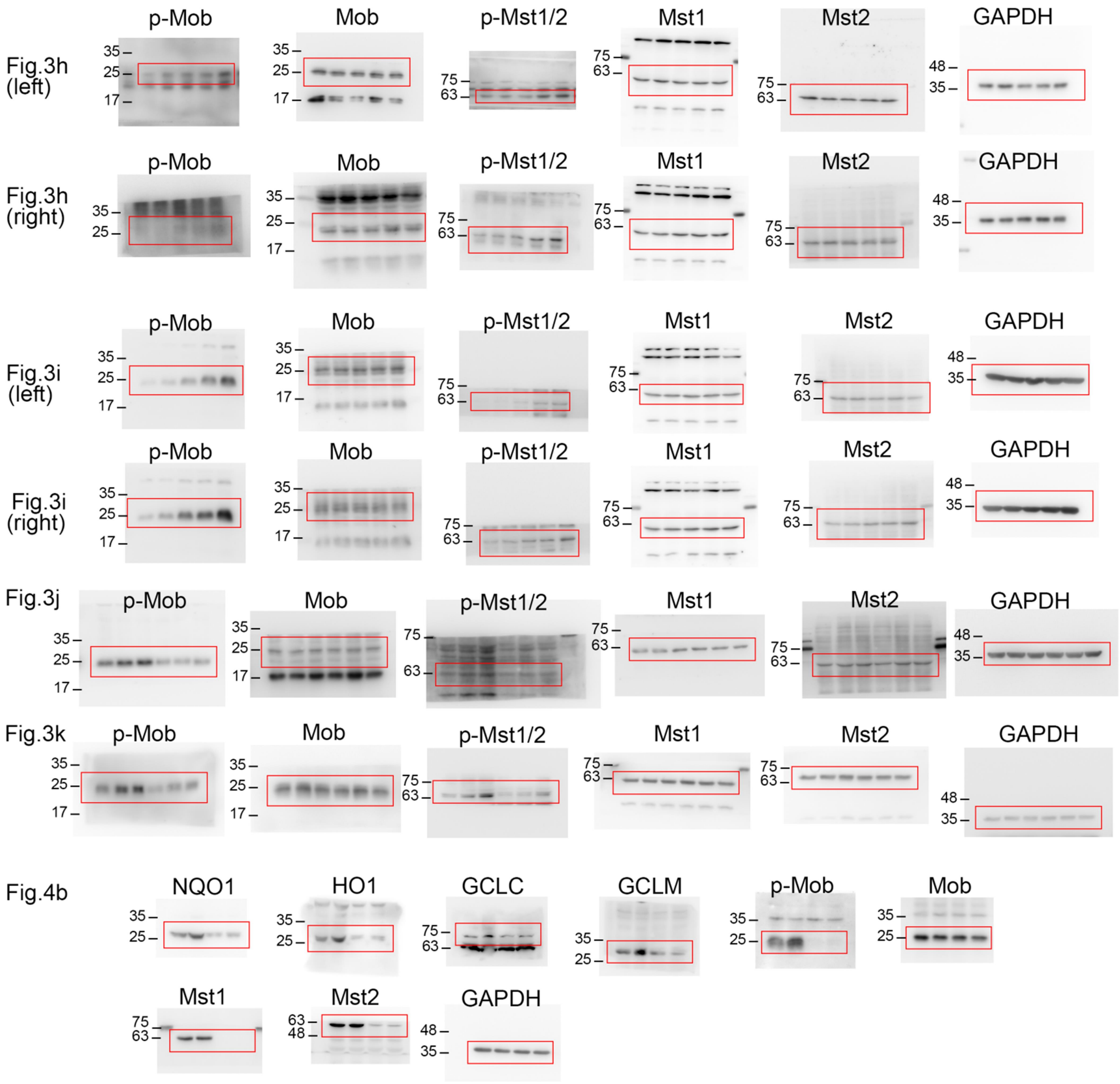

Supplementary Figure 6. Full scans for Fig. 3i-k and 4b.

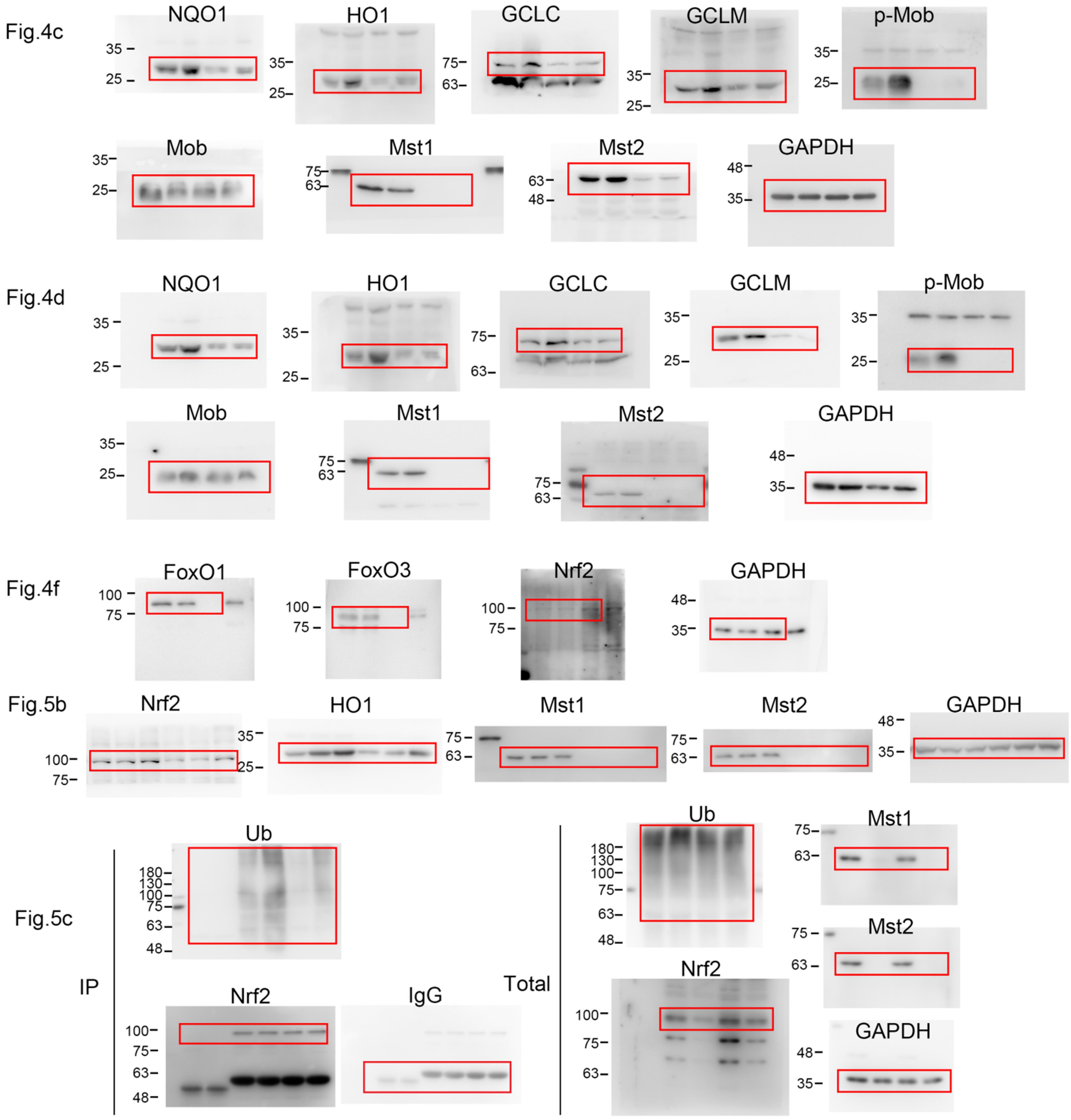

Supplementary Figure 7. Full scans for Fig. 4c, 4d, 4f, 5b and 5c.

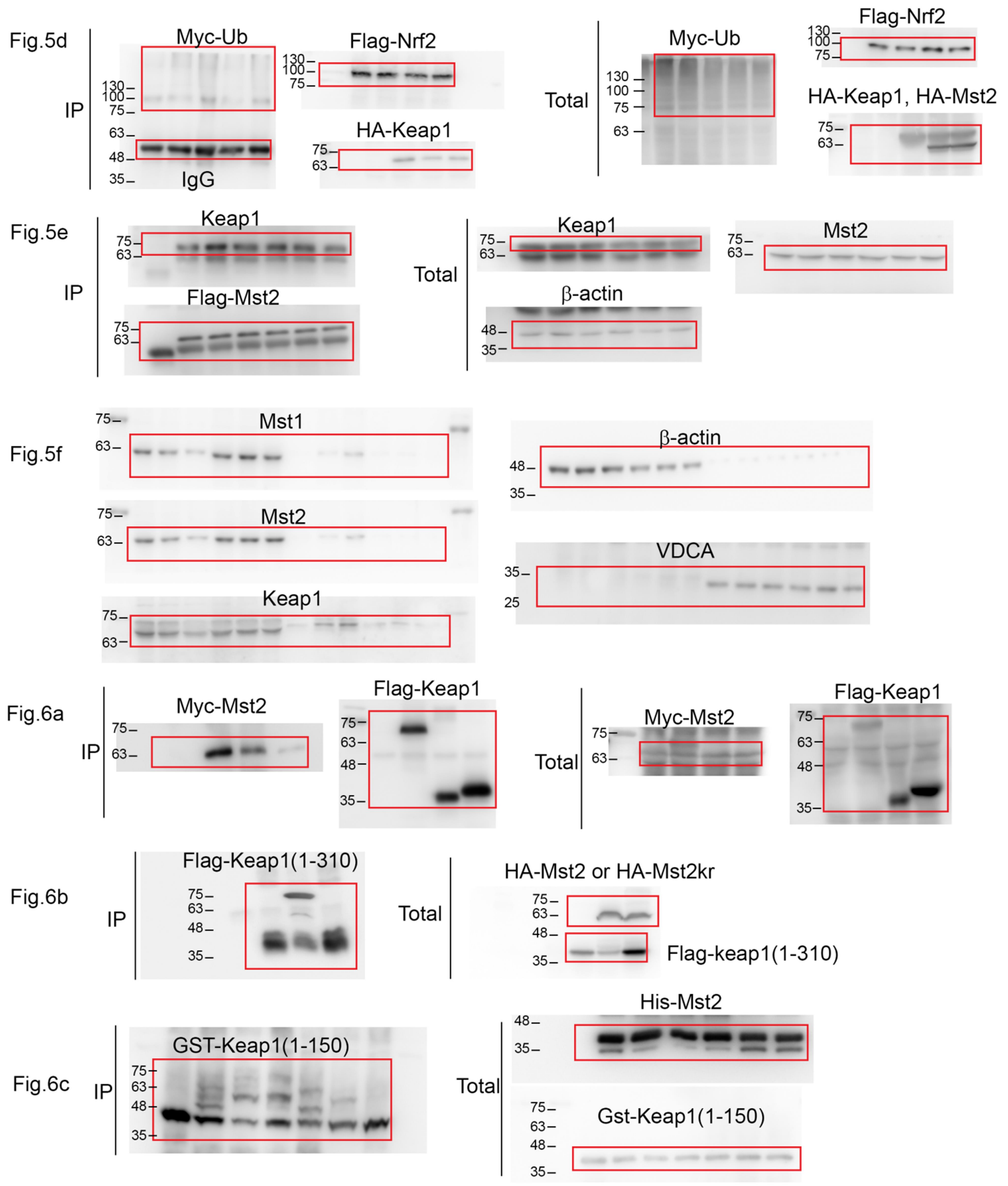

Supplementary Figure 8. Full scans for Fig. 5d-f, 6a-c.

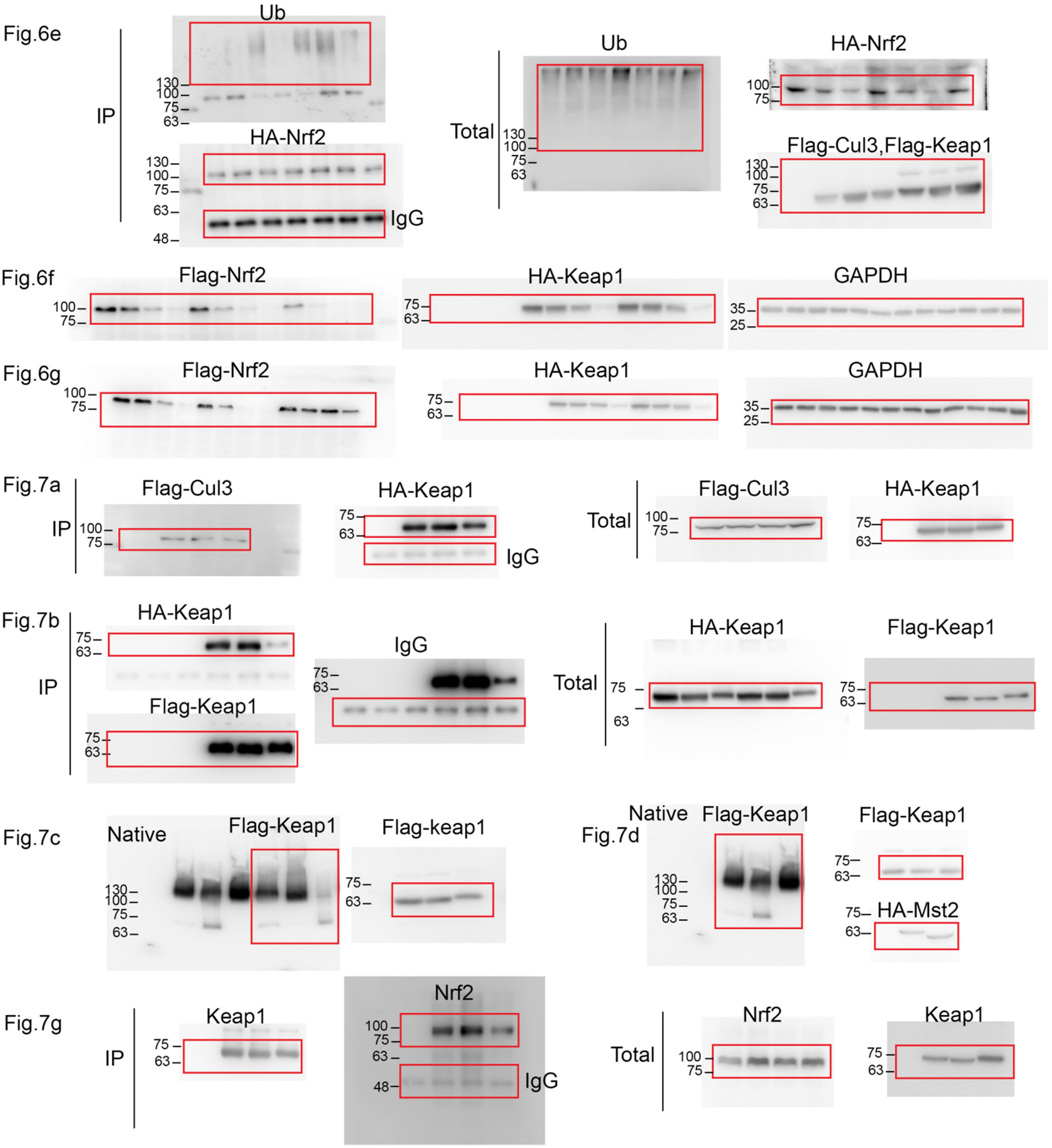

Supplementary Figure 9. Full scans for Fig. 6e-g, 7a-d, g.

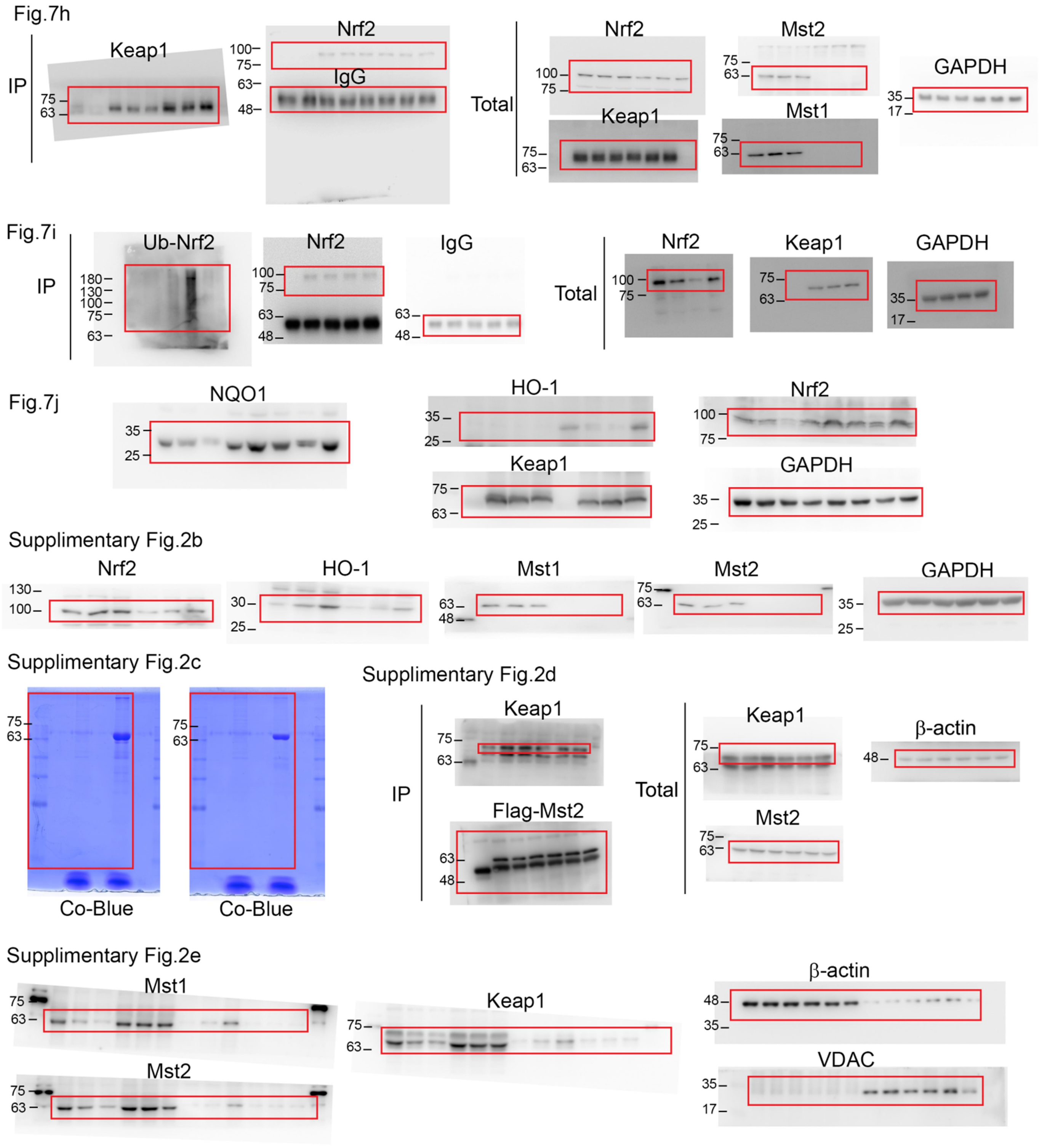

Supplementary Figure 10. Full scans for Fig. 7h-j and Supplementary Fig.2b-e.

Supplementary Table 1. The sequence of primers used on the study

Species: h.—Human; m.—Mouse

Gene name	Forward primer sequence(5'-3')	Reverse primer sequence(5'-3')	Restriction enzyme
h. *MST1*	AAAAAAGCTTATGGGGTGGCT CCCACTCC	AAAACTCGAGCTAACCCAGTCTCAT GACCTTG	HindIII & XhoI
h. *MST2*	AAAAGGTACCATGGAGCAGCC GCCGGCGCC	AAAACTCGAGTCAAAAGTTTTGCTG CCTTC	KpnI & XhoI
h. *NRF2*	ATGATGGACTTGGAGCTGCC	CTAGTTTTTCTTAACATCTG	
h. *KEAP1*	ATGCAGCCAGATCCCAGGCC	TCAACAGGTACAGTTCTGC	
h. *KEAP1* (1-150)	ATGCAGCCAGATCCCAGGCC	TCACTTCTCGCCCATGGAGATGG	
h. *KEAP1* (1-310)	ATGCAGCCAGATCCCAGGCC	TCACAGGGTGAGCTCCTCGAAG	
h. *KEAP1* (311-624)	ATGCACAAGCCCACGCAGGTG	TCAACAGGTACAGTTCTGC	
h. *CUL3*	ATGTCGAATCTGAGCAAAGG	TTATGCTACATATGTGTATAC	
Ad-*KEAP1*	CGTCGACGGAGATCTATGC AGCCAGATCCCAGG	CGCAAGCTTCTCGAGTCAACAGGT ACAGTTCTGC	BglII & XhoI
Ad-*NRF2*	CGTCGACGGAGATCTATGA TGGACTTGGAGCTGC	CGCAAGCTTCTCGAGCTAGTTTTT CTTAACATC	BglII & XhoI

qPCR primers	Forward primer sequence(5'-3')	Reverse primer sequence(5'-3')
m. *Gclc*	GGGGTGACGAGGTGGAGTA	GTTGGGGTTTGTCCTCTCCC
m. *Gclm*	AGGAGCTTCGGGACTGTATCC	GGGACATGGTGCATTCCAAAA
m. *Ho-1*	AAGCCGAGAATGCTGAGTTCA	GCCGTGTAGATATGGTACAAGGA
m. *Nqo1*	AGGATGGGAGGTACTCGAATC	AGGATGGGAGGTACTCGAATC
m. *Sod1*	AACCAGTTGTGTTGTCAGGAC	CCACCATGTTTCTTAGAGTGAGG
m. *Sod2*	CAGACCTGCCTTACGACTATGG	CTCGGTGGCGTTGAGATTGTT
m. *Cat*	AGCGACCAGATGAAGCAGTG	TCCGCTCTCTGTCAAAGTGTG
m. *Foxo1*	CCCAGGCCGGAGTTTAACC	GTTGCTCATAAAGTCGGTGCT
m. *Foxo3*	CTGGGGGAACCTGTCCTATG	TCATTCTGAACGCGCATGAAG
m. *Nrf2*	CTTTAGTCAGCGACAGAAGGAC	AGGCATCTTGTTTGGGAATGTG
m. *Gapdh*	AGGTCGGTGTGAACGGATTTG	TGTAGACCATGTAGTTGAGGTCA
Telomeric primer	CGGTTTGTTTGGGTTTGGGTTTGGGTTT GGGTTTGGGTT	GGCTTGCCTTACCCTTACCCTTAC CCTTACCCTTACCCT
m. 36B4	ACTGGTCTAGGACCCGAGAAG	TCAATGGTGCCTCTGGAGATT

Others	Forward primer sequence(5'-3')	Reverse primer sequence(5'-3')
h. *KEAP1*	CATGGCAACCGCGCCTTCAGCTAC	GTAGCTGAAGGCGCGGTTGCCATG

(T51A)		
h. *KEAP1* (S53A)	CGCACCTTCGCCTACACCCTGGAG	CTCCAGGGTGTAGGCGAAGGTGCG
h. *KEAP1* (T55A)	ACCTTCAGCTACGCCCTGGAGGATCAT	ATGATCCTCCAGGGCGTAGCTGAAGGT
h. *KEAP1* (T80A)	CTGTGTGACGTCGACCTGCAGGTCAAG	CTTGACCTGCAGTGCGACGTCACACAG
h. *KEAP1* (T51D)	ATGGCAACCGCGACTTCAGCTACACC	GGTGTAGCTGAAGTCGCGGTTGCCAT
h. *KEAP1* (S53D)	AACCGCACCTTCGACTACACCCTGGAG	CTCCAGGGTGTAGTCGAAGGTGCGGTT
h. *KEAP1* (T55D)	ACCTTCAGCTACGACCTGGAGGATCAT	ATGATCCTCCAGGTCGTAGCTGAAGGT
h. *KEAP1* (T80D)	CTGTGTGACGTCGACCTGCAGGTCAAG	CTTGACCTGCAGGTCGACGTCACACAG
