# Supplementary material for: Macrophage achieves self-protection against oxidative stress-induced ageing through the Mst-Nrf2 axis
Source: Nat Commun. 2019 Feb 14;10:755. doi: 10.1038/s41467-019-08680-6 (PMC6376064; doi:10.1038/s41467-019-08680-6)
Supplement: Supplementary file 3 — Reporting Summary [file 41467_2019_8680_MOESM3_ESM.pdf]

nature research

Corresponding author(s): Lanfen Chen

Reporting Summary

Nature Research wishes to improve the reproducibility of the work that we publish. This form provides structure for consistency and transparency in reporting. For further information on Nature Research policies, see Authors & Referees and the Editorial Policy Checklist.

Statistical parameters

When statistical analyses are reported, confirm that the following items are present in the relevant location (e.g. figure legend, table legend, main text, or Methods section).

n/a	Confirmed	
☐	☒	The exact sample size (n) for each experimental group/condition, given as a discrete number and unit of measurement
☐	☒	An indication of whether measurements were taken from distinct samples or whether the same sample was measured repeatedly
☐	☒	The statistical test(s) used AND whether they are one- or two-sided<br>*Only common tests should be described solely by name; describe more complex techniques in the Methods section.*
☒	☐	A description of all covariates tested
☒	☐	A description of any assumptions or corrections, such as tests of normality and adjustment for multiple comparisons
☐	☒	A full description of the statistics including central tendency (e.g. means) or other basic estimates (e.g. regression coefficient) AND variation (e.g. standard deviation) or associated estimates of uncertainty (e.g. confidence intervals)
☒	☐	For null hypothesis testing, the test statistic (e.g. F, t, r) with confidence intervals, effect sizes, degrees of freedom and P value noted<br>*Give P values as exact values whenever suitable.*
☒	☐	For Bayesian analysis, information on the choice of priors and Markov chain Monte Carlo settings
☒	☐	For hierarchical and complex designs, identification of the appropriate level for tests and full reporting of outcomes
☒	☐	Estimates of effect sizes (e.g. Cohen's d, Pearson's r), indicating how they were calculated
☐	☒	Clearly defined error bars<br>*State explicitly what error bars represent (e.g. SD, SE, CI)*

Our web collection on statistics for biologists may be useful.

Software and code

Policy information about availability of computer code

Data collection	N/A
Data analysis	Prism6 GraphPad www.graphpad.com/scientificsoftware/pri-sm<br>FlowJo software (TreeStar)<br>ImageJ https://imagej.nih.gov/ij<br>ProteinPilot Proteinpilot Version 4.5

For manuscripts utilizing custom algorithms or software that are central to the research but not yet described in published literature, software must be made available to editors/reviewers upon request. We strongly encourage code deposition in a community repository (e.g. GitHub). See the Nature Research guidelines for submitting code & software for further information.

April 2018

Data

Policy information about availability of data

All manuscripts must include a data availability statement. This statement should provide the following information, where applicable:

- Accession codes, unique identifiers, or web links for publicly available datasets
- A list of figures that have associated raw data
- A description of any restrictions on data availability

All data generated or analysed during this study are included in this manuscript (and its supplementary information files).

Field-specific reporting

Please select the best fit for your research. If you are not sure, read the appropriate sections before making your selection.

☒ Life sciences ☐ Behavioural & social sciences ☐ Ecological, evolutionary & environmental sciences

For a reference copy of the document with all sections, see nature.com/authors/policies/ReportingSummary-flat.pdf

Life sciences study design

All studies must disclose on these points even when the disclosure is negative.

Sample size	Sample size was determined accordingly to previous published study and experimental knowledge.
Data exclusions	No data were excluded from the experiments reported.
Replication	Experimental findings were highly reproducible each time.
Randomization	The age and gender matched animals were randomized into control and treated groups
Blinding	The investigators were blinded to allocation during experiments and outcome assessment.

Reporting for specific materials, systems and methods

Materials & experimental systems

n/a	Involved in the study	
☐	☒	Unique biological materials
☐	☒	Antibodies
☐	☒	Eukaryotic cell lines
☒	☐	Palaeontology
☐	☒	Animals and other organisms
☒	☐	Human research participants

Methods

n/a	Involved in the study	
☒	☐	ChIP-seq
☐	☒	Flow cytometry
☒	☐	MRI-based neuroimaging

Unique biological materials

Policy information about availability of materials

Obtaining unique materials	All established cellular and plasmid reagents are available on reasonable request.

Antibodies

Antibodies used

Phospho-Histone H2A.X (Ser139) (20E3) Rabbit mAb (Cell Signaling Technology) cat#9718: WB IF
Histone H2A (L88A6) Mouse mAb (Cell Signaling Technology)cat#3636 :WB
Cleaved Caspase-3 (Asp175) (5A1E) Rabbit mAb (Cell Signaling Technology)cat#9664 : WB
MST1 Antibody (Cell Signaling Technology)cat#3682 WB
MST2 Antibody(Cell Signaling Technology) cat#3952 WB
GAPDH (D16H11) XP® Rabbit mAb(Cell Signaling Technology) cat#5174 WB

FoxO1 (C29H4) Rabbit mAb (Cell Signaling Technology) cat#2880 WB IF
FoxO3a (D19A7) Rabbit mAb (Cell Signaling Technology) cat#12829 WB
α-Tubulin Antibody(Cell Signaling Technology) cat#2144 WB
Ubiquitin (P4D1) Mouse mAb(Cell Signaling Technology) cat#3936 WB
NRF2 (D1Z9C) XP® Rabbit mAb (Cell Signaling Technology) cat#12721 IP
Normal Rabbit IgG (Cell Signaling Technology) cat #2729 IP
VVDAC antibody Rabbit monoclonal Abcam Cat: #4661
DYKDDDDK Tag (D6W5B) Rabbit mAb (Binds to same epitope as Sigma's Anti-FLAG® M2 Antibody) (Cell Signaling Technology) cat #14793 WB IF
Phospho-MOB1 (Thr35) (D2F10) Rabbit mAb Cell Signaling Technology Cat#8699 WB
MOB1 (E1N9D) Rabbit Cell Signaling Technology Cat#13730 WB
PARP1 Antibody Rabbit Polyclonal proteintech Cat: 13371-1-AP WB
KEAP1 Antibody Rabbit Polyclonal proteintech Cat: 10503-2-AP WB
HSP60 Antibody Mouse Polyclonal proteintech Cat: 66041-1-lg WB
STK4 Antibody Rabbit Polyclonal proteintech Cat: 22245-1-AP IF
GST Antibody Mouse Polyclonal proteintech Cat: 66001-2-Ig WB
HA-probe (F-7) antibody Mouse Polyclonal Santa cruz cat: sc-7392 WB
HA-probe (Y-11) antibody Rabbit Polyclonal Santa cruz cat: sc-805 IF
MYC-probe (F-7) antibody Mouse Polyclonal Santa cruz cat: sc-40 WB IF
Nrf2 (EP1808Y) antibody Rabbit monoclonal Abcam Cat: ab62352 WB
Nrf2 antibody Rabbit polyclonal Abcam Cat: ab31163 IF
TOMM20 antibody Mouse monoclonal Abcam Cat: ab56783 IF
VDAC antibody Rabbit monoclonal Abcam Cat: #4661

Validation

Phospho-Histone H2A.X (Ser139) (20E3) Rabbit mAb (Cell Signaling Technology cat#9718):Western blot analysis of extracts from untreated or UV-treated 293 cells and Confocal immunofluorescent analysis of HeLa cells, (see manufacturer's website).
Histone H2A (L88A6) Mouse mAb (Cell Signaling Technology)cat#3636:Western blot analysis of extracts from various cell lines (see manufacturer's website).
Cleaved Caspase-3 (Asp175) (5A1E) Rabbit mAb (Cell Signaling Technology)cat#9664:Western blot analysis of extracts from C6 (rat), NIH/3T3 (mouse), and Jurkat (human) cells (see manufacturer's website).
MST1 Antibody (Cell Signaling Technology)cat#3682:Western blot analysis of extracts from cos-7 cell lines (see manufacturer's website).
MST2 Antibody(Cell Signaling Technology) cat#3952: Western blot analysis of extracts from c6 cell lines (see manufacturer's website).
GAPDH (D16H11) XP® Rabbit mAb(Cell Signaling Technology) cat#5174; Western blot analysis of extracts from hela cell lines (see manufacturer's website).
FoxO1 (C29H4) Rabbit mAb (Cell Signaling Technology) cat#2880: Western blot analysis of extracts from IGROV-1 and COS-7 cells, Confocal immunofluorescent analysis of IGROV-1 cells (see manufacturer's website).
FoxO3a (D19A7) Rabbit mAb (Cell Signaling Technology) cat#12829: Western blot analysis of extracts from 293T, MRK-nu-1 and Jurkat cells(see manufacturer's website).
α-Tubulin Antibody(Cell Signaling Technology) cat#2144: Western blot analysis of extracts from CAD and C6 cells, (see manufacturer's website).
Ubiquitin (P4D1) Mouse mAb(Cell Signaling Technology) cat#3936: Western blot analysis of 293 and HeLa cells, untreated or treated with the 26S proteasome inhibitor MG132(see manufacturer's website).
NRF2 (D1Z9C) XP® Rabbit mAb (Cell Signaling Technology) cat#12721: Immunoprecipitation of NRF2 from MEF wt cell extracts treated with MG-132(see manufacturer's website)
Normal Rabbit IgG (Cell Signaling Technology) cat #2729;Immunoprecipitation of 4E-BP2 from C2C12 cell extracts (see manufacturer's website).
DYKDDDDK Tag (D6W5B) Rabbit mAb (Binds to same epitope as Sigma's Anti-FLAG® M2 Antibody) (Cell Signaling Technology) cat #14793: Confocal immunofluorescent analysis of 293T cells transfected with a GFP-DYKDDDDK-Tag (see manufacturer's website).
Phospho-MOB1 (Thr35) (D2F10) Rabbit mAb （CST，Cat#8699）: Western blot analysis of extracts from MCF7 cells (see manufacturer's website).
MOB1 (E1N9D) Rabbit (CST, Cat#13730): Western blot analysis of extracts from various cell lines (see manufacturer's website).
PARP1 Antibody Rabbit Polyclonal proteintech Cat: 13371-1-AP: Western blot analysis of extracts from C6, A549 and Jurkat cells (see manufacturer's website).
KEAP1 Antibody Rabbit Polyclonal proteintech Cat: 10503-2-AP: Western blot analysis of extracts from Jurkat cells (see manufacturer's website).
HSP60 Antibody Mouse Polyclonal proteintech Cat: 66041-1-lg : Western blot analysis of extracts from RAW 264.7cells (see manufacturer's website).
STK4 Antibody Rabbit Polyclonal proteintech Cat: 22245-1-AP: Confocal immunofluorescent analysis of HepG2 cells (see manufacturer's website).
GST Antibody Mouse Polyclonal proteintech Cat: 66001-2-Ig: Western blot analysis of GST protein from E.coli (see manufacturer's website).
HA-probe (F-7) antibody Mouse Polyclonal Santa cruz cat: sc-7392: Western blot analysis of HA-LacZ fusion protein expression in COS cells (see manufacturer's website).
HA-probe (Y-11) antibody Rabbit Polyclonal Santa cruz cat: sc-805: same clone was used in Manuele Rebsamen et.al Nature. 2015 March 26; 519(7544): 477–481. doi:10.1038/nature14107.
Western blot analysis of c-HA expression in 293T and Immunofluorescence staining of methanol-fixed COS cells transfected with c-Myc fusion protein(see manufacturer's website).
MYC-probe (F-7) antibody Mouse Polyclonal Santa cruz cat: sc-40 : Western blot analysis of c-Myc expression in HeLa and Immunofluorescence staining of methanol-fixed COS cells transfected with c-Myc fusion protein(see manufacturer's website).
Nrf2 (EP1808Y) antibody Rabbit monoclonal Abcam Cat: ab62352: Western blot analysis of extracts from HeLa, Saos-2 and THP-1 cells (see manufacturer's website).
Nrf2 antibody Rabbit polyclonal Abcam Cat: ab31163: Confocal immunofluorescent analysis of HeLa cells (see manufacturer's website).

TOMM20 antibody Mouse monoclonal Abcam Cat: ab56783: Confocal immunofluorescent analysis of HeLa cells (see manufacturer's website).
VDAC antibody Rabbit monoclonal Abcam Cat: #4661: Immunoblot analysis of WT BMDMs (see manufacturer's website).

Eukaryotic cell lines

Policy information about cell lines

Cell line source(s): Mouse BMDMs and peritoneal macrophages were isolated from mouse bone marrow and peritoneal cavity respectively . The 293T, HeLa, RAW264.7 and THP1 cell lines were originally obtained from the American Type Culture Collection.

Authentication: All cells were originally obtained from the ATCC cell repository.

Mycoplasma contamination: All cells were tested for mycoplasma contamination and were found to be negative

Commonly misidentified lines (See ICLAC register): No commonly misidentified cell lines were used.

Animals and other organisms

Policy information about studies involving animals; ARRIVE guidelines recommended for reporting animal research

Laboratory animals: All mice were maintained under specific pathogen-free conditions at the Xiamen University Labortory Animal Center. These mouse experiments were approved by the Institutional Animal Care and Use Committee and were in strict accordance with good animal practice as defined by the Xiamen University Laboratory Animal Center.
Wild-type C57BL/6, Lyz2-Cre mice (004781) and B6.129X1-Nfe2l2tm1Ywk/J (Nrf2-/-, 017009), Foxo1tm1Rdp/J (Foxo1fl/fl, 024756), Foxo3tm1Rdp/J (Foxo3fl/fl, 024668) were originally from the Jackson Laboratory.
Mst1fl/fll mice were from Dr. R. L. Johnson's lab, University of Texas, M.D. Anderson Cancer Center, Houston, USA
Mst2fl/fl mouse was described in Zhou D, et al, Cancer Cell. 2009 Nov 6;16(5):425-38.

Wild animals: The study does not involve wild animals

Field-collected samples: The study does not involve field-collected samples

Flow Cytometry

Plots

Confirm that:

☒ The axis labels state the marker and fluorochrome used (e.g. CD4-FITC).

☒ The axis scales are clearly visible. Include numbers along axes only for bottom left plot of group (a 'group' is an analysis of identical markers).

☒ All plots are contour plots with outliers or pseudocolor plots.

☒ A numerical value for number of cells or percentage (with statistics) is provided.

Methodology

Sample preparation: Single cells isolated from the bone marrow, spleen or peritoneal cavity were stained for 30 min with the appropriate fluorescence-conjugated antibodies and washed, then were resuspended with flow cytometry staining buffer (2% FBS in PBS) containing DAPI (4′,6-diamidino-2-phenylindole; Invitrogen).
Cells (BMDMs) were plated in non–tissue-culture-treated dishes. Samples were treated with NAC (5 μM) for 30 min as needed, followed by the treatment of other indicated stimulants (10 μM antimycin A or 3 μM rotenone). The culture medium was removed and then the cells were washed with PBS and then incubated for 30 min at 37℃with MitoSOX (for measurement of mROS superoxide; Invitrogen) and/or CM-H2DCFDA or CellROX (for measurement of total cellular H2O2; Invitrogen) at a final concentration of 5 μM in serum-free DMEM (Invitrogen). The cells were washed with warmed PBS, removed from the plates by pipetting with 1% trypsin containing 1 mM EDTA, pelleted at 1,600 r.p.m. for 3 min, immediately re-suspended in cold PBS containing 1% FBS and analyzed by flow cytometry.

Instrument: BD LSRFortessa flow cytometer (BD Biosciences)

Software: Data were collected with BD FACSDIVA™ SOFTWARE and analyzed with FlowJo software (TreeStar)

Cell population abundance: More than 95%

Gating strategy: Titration of the antibodies was performed to determine the optimal concentration of the antibodies for staining of cells. FMO (Flourescence Minus One) assay were used to determine fluorochrome overlap and set negative gate.
Unstained cells were used to set gates. Single antibody stained cells were used to define positive gates.

☒ Tick this box to confirm that a figure exemplifying the gating strategy is provided in the Supplementary Information.
